# Structural Characterization and Biological Properties Analysis of Exopolysaccharides Produced by *Weisella cibaria* HDL-4

**DOI:** 10.3390/polym16162314

**Published:** 2024-08-15

**Authors:** Bosen Zhou, Changli Wang, Yi Yang, Wenna Yu, Xiaoyun Bin, Gang Song, Renpeng Du

**Affiliations:** 1 Engineering Research Center of Agricultural Microbiology Technology, Ministry of Education & Heilongjiang Provincial Key Laboratory of Plant Genetic Engineering and Biological Fermentation Engineering for Cold Region & Key Laboratory of Microbiology, College of Heilongjiang Province & School of Life Sciences, Heilongjiang University, Harbin 150080, China; 2221609@s.hlju.edu.cn (B.Z.); 2221595@s.hlju.edu.cn (Y.Y.); 2231660@s.hlju.edu.cn (W.Y.); 2 College of Basic Medical Sciences, Youjiang Medical University for Nationalities, Baise 533000, China; 01111@ymun.edu.cn (C.W.); xiaoyun_bin@ymcn.edu.cn (X.B.); 3 State Key Laboratory of Microbial Metabolism, School of Life Sciences and Biotechnology, Shanghai Jiao Tong University, Shanghai 200240, China

**Keywords:** *Weissella cibaria*, exopolysaccharide, characterization, purification, application

## Abstract

An exopolysaccharide (EPS)-producing strain, identified as *Weissella cibaria* HDL-4, was isolated from litchi. After separation and purification, the structure and properties of HDL-4 EPS were characterized. The molecular weight of HDL-4 EPS was determined to be 1.9 × 10⁶ Da, with glucose as its monosaccharide component. Fourier transform infrared spectroscopy (FT-IR) and nuclear magnetic resonance (NMR) analyses indicated that HDL-4 EPS was a D-glucan with α-(1→6) and α-(1→4) glycosidic bonds. X-ray diffraction (XRD) analysis revealed that HDL-4 EPS was amorphous. Scanning electron microscope (SEM) and atomic force microscope (AFM) observations showed that HDL-4 EPS possesses pores, irregular protrusions, and a smooth layered structure. Additionally, HDL-4 EPS demonstrated significant thermal stability, remaining stable below 288 °C. It exhibited a strong metal ion adsorption activity, emulsification activity, antioxidant activity, and water-retaining property. Therefore, HDL-4 EPS can be extensively utilized in the food and pharmaceutical industries as an additive and prebiotic.

## 1. Introduction

Lactic acid bacteria (LAB) exopolysaccharides (EPSs) are secreted by LAB during growth metabolism and accumulate outside the cell walls as long-chain, high-molecular-weight linear or branched carbohydrate polymers [1]. LAB that produce EPS primarily include *Leuconostoc*, *Streptococcus*, *Weissella*, *Lactobacillus*, *Pseudomonas*, and *Bifidobacterium* [2]. Microbial cell wall-bound EPS is known as capsular exopolysaccharide (C-EPS), while the mucous EPS released into the culture medium is termed slime exopolysaccharide (S-EPS) [3]. Additionally, based on their monosaccharide composition, these EPSs are categorized into two groups: homopolysaccharides (HoPSs), composed solely of one type of monosaccharide, and heteropolysaccharides (HePSs), which contain multiple types of monosaccharides [4]. Understanding the structural characteristics of LAB EPS is fundamental to studying their physical, chemical functions, and biological activities. The chemical structure of EPS varies significantly among different LAB strains [5,6]. LAB EPS was a biopolymer, and the molecular weight of most LAB EPSs ranged from 4 × 10⁴ to 6 × 10⁶ Da. Previous studies have shown that the physical and chemical properties of LAB EPS were influenced by its structure, molecular weight, monosaccharide composition, glycosidic bonds, and surface morphology. These properties confer numerous advantageous characteristics, making LAB EPS widely applicable in the food, pharmaceutical, and other industrial sectors [6,7].

In recent years, probiotics and their fermented products have become increasingly favored by consumers. LAB, as a significant probiotic, has become synonymous with health and wellness. Furthermore, as a recognized green and safe food-grade microorganism (GRAS), the metabolite EPS produced by LAB has garnered significant scholarly attention due to its excellent properties [8]. As a secondary metabolite of LAB, LAB EPS is a natural, nontoxic, biodegradable, and environmentally friendly polymer with anti-tumor, antiviral, antioxidant, and hypoglycemic functions [9]. Additionally, LAB EPS is renowned for its functional characteristics in food, cosmetics, and dairy products, as well as its ability to extend shelf life and provide health benefits [10]. It has been reported that LAB EPS imparts excellent viscosity and rheological properties, making it a valuable food additive in fermented dairy products, where it significantly improves food stability, rheological properties, and sensory quality [11]. For example, glucans, produced by *Leuconostoc*, *Weissella,* and *Lactobacillus* species, were mainly used as indirect food additives, stabilizers, and viscosity agents [12]. The research conducted by Brüls et al. [13] confirmed that EPS produced by the *Lactococcus* lactis strain can enhance the sensory characteristics of yogurt. Additionally, LAB EPS can be utilized to customize yogurt in terms of microstructure, rheological properties, and resistance to dehydration shrinkage. Studies have shown that LAB EPS can replace antibiotics to regulate intestinal health and improve the immune system [14]. EPS produced by *Lactobacillus buchneri* can improve the intestinal flora imbalance and adjust lipopolysaccharide/D-galactosamine-induced liver injury. Therefore, LAB EPS can be widely utilized in various fields as functional products such as food additives, health care products, and medical materials.

The functionality and attributes of EPS are intrinsically linked to its structural composition. To fully harness its potential and expand its use in sectors such as food and pharmaceuticals, understanding the relationship between EPS’s structure and function is essential. This research focused on isolating *Weissella cibaria* HDL-4, a strain prolific in EPS production from litchi, and subsequently examining the structural composition and physicochemical properties of EPS. These findings provide foundational knowledge for the exploitation of EPS in diverse industrial applications.

## 2. Materials and Methods

### 2.1. Isolation and Identification of Strains

The initial selection of LAB capable of producing EPS was conducted using litchi samples on MRS agar plates supplemented with 2% sucrose. Individual 50 g litchi samples from Yunnan, China, were mixed with 200 mL sterile water in a hermetical fermenter and fermented at 20 °C for 72 h. Subsequently, the samples were serially diluted, then the dilutions were applied to MRS-S medium at 30 °C for 48 h. The formation of mucoid substances secreted by the candidate strains after incubation was observed, indicating that an EPS-positive signal was generated. The EPS-producing strain was preserved in MRS broth mixed with 40% glycerol and stored at −80 °C. Identification of the bacterium at the family, genus, and species levels was performed by examining its morphological characteristics and conducting 16S rRNA sequencing analysis. The PCR amplification and sequencing procedures were outsourced to the professional services of Shenggong (Shanghai). Subsequently, the generated sequences were aligned using NCBI’s nucleotide BLAST and compared against the GenBank database containing authenticated prokaryotic strains. For evolutionary analysis, MEGA 11 software was utilized, employing the maximum-likelihood algorithm.

### 2.2. Isolation and Purification of HDL-4 EPS

Following the methodology outlined by Yu et al. [15] with minor adjustments, the extraction, isolation, and purification of EPS proceeded as follows. The cultivation of *W. cibaria* HDL-4 occurred at 30 °C for 24 h in MRS medium. Subsequently, a 100 mL portion of the culture was transferred to 900 mL of MRS-S medium, where glucose was replaced with 20 g/L sucrose. This fresh mixture underwent fermentation in a shaker incubator for 36 h. The contents were then centrifuged (12,000 rpm, 20 min, 4 °C), and the supernatant was collected. Next, the supernatant was mixed with three times its volume of 95% ethanol (*v*/*v*) and stored at 4 °C overnight. A second centrifugation (12,000 rpm, 20 min, 4 °C) followed, and the precipitate was dissolved in sterile distilled water. An equal volume of 10% trichloroacetic acid (*w*/*v*) was added, and the mixture was refrigerated at 4 °C for 10 h. This process led to the crude EPS (C-EPS) extraction via centrifugation (12,000 rpm, 40 min, 4 °C). The C-EPS was then placed in a 10 kDa dialysis membrane and dialyzed against distilled water at 4 °C for two days, with the water being refreshed twice daily. For purification, the C-EPS underwent gel filtration chromatography on a Sephadex G-100 column, using deionized water as the mobile phase at a flow rate of 0.2 mL/min. The eluted fraction was freeze-dried to yield the purified EPS (P-EPS). The total sugar content of the EPS was quantified using the phenol-sulfuric acid method [16].

### 2.3. Purity of HDL-4 EPS

The purified HDL-4 EPS was dissolved in deionized water to 1 mg/mL. A UV-Visible spectrophotometer (SP-1920UV, Spectrum Instruments, Shanghai, China) was used to evaluate the purity of HDL-4 EPS within a spectral range of 190 to 400 nm. Protein content was assayed by the Bradford method [17]. The elemental composition, including the contents of carbon, hydrogen, nitrogen, and sulfur, of HDL-4 EPS was determined using an elemental analyzer (Vario Micro cube, Elementar, Germany) [18].

### 2.4. Monosaccharide Composition of HDL-4 EPS

Monosaccharide composition was conducted using gas chromatography (GC) with an Agilent 6280 system from Agilent Technologies, USA, following the procedure described by Yang et al. [17]. Individual 10 mg EPS samples were hydrolyzed in 2 mL of 2 M TFA at a temperature of 120 °C for 6 h. TFA was removed by evaporation. After mixing with acetic anhydride, acetylation was carried out at 90 °C for 30 min, and the derivatization process was completed. The monosaccharides were identified by comparing their retention times with those of standard sugar references.

### 2.5. Molecular Weight of HDL-4 EPS

The molecular weight (Mw) of HDL-4 EPS was determined via gel permeation chromatography (GPC). Initially, the lyophilized EPS was reconstituted in ultrapure water to a concentration of 1 g/L. Subsequently, a 100 μL aliquot of this solution was analyzed using a TSKgel chromatographic column, maintained at 27 °C. The column was supplied by the Tosoh Corporation, Tokyo, Japan. Elution was carried out with a 100 mmol/L NaNO_3_ solution at a flow rate of 0.8 mL/min, and the process was monitored by a Refractometer RI-150 from Spectra Systems (Thermo Fisher Scientific, Waltham, MA, USA). The MW was calculated using a calibration curve derived from various standard dextran benchmarks (1000, 500, 650, and 50 kDa) obtained from Sigma-Aldrich (St. Louis, MO, USA).

### 2.6. Fourier-Transform Infrared (FT-IR) Spectroscopy Analysis

The infrared spectrum of HDL-4 EPS was captured using an FT-IR spectrometer (Bruker FTS3000, Ettlingen, Germany), operating within a spectral range of 400 to 4000 cm^−1^. In a moisture-free setting, a small quantity of EPS was combined with anhydrous potassium bromide powder in a pestle and mortar. The mixture was then finely ground and compressed into a transparent pellet using a tablet press. Prior to sample analysis, the system first recorded the background, followed by the infrared spectra of the EPS samples.

### 2.7. X-ray Diffraction (XRD) Analysis

An X-ray diffractometer (X’pert PRO MPD, Panalytical, Almelo, The Netherlands) was used to analyze the crystal structure of HDL-4 EPS at a 2θ angle range of 10–80°, with a scanning rate of 2°/min.

### 2.8. Nuclear Magnetic Resonance (NMR) Spectroscopy Analysis

The purified EPS samples were dissolved in HDO to reach a final concentration of 20 mg/mL EPS solution. Then, 1D NMR (^1^H NMR, ^13^C NMR) and 2D NMR (COSY, HSQC) were measured using a Bruker Avance III spectrometer (Bruker, Bremen, Germany) at room temperature. Three exchanges were conducted to eliminate the remaining solvent containing protons. Afterwards, transfer to a nuclear magnetic tube was performed followed by testing. Analysis was carried out at 25 °C using 400 MHz. HDO was used as an internal standard. Chemical shifts were measured in ppm.

### 2.9. Scanning Electron Microscopy (SEM) and Atomic Force Microscopy (AFM) Analysis

The purified HDL-4 EPS was freeze-dried and adhered to conductive adhesive for surface morphology analysis using SEM (Hitachi, Chiyoda City, Japan). A 5 μL volume from a solution of EPS at a concentration of 1 mg/mL was applied onto pristine mica surfaces and evaporated under a gentle stream of N_2_ gas. The three-dimensional architecture and molecular shape of the EPS specimens were then examined using AFM equipment provided by Bruker (Bremen, Germany).

### 2.10. Water Contact Angle Measurement

The assessment of HDL-4 EPS’s water contact angle was executed adopting the technique detailed by Jiang et al. [19] The measurements were recorded utilizing a contact angle analyzer (model JY-82B, sourced from Chengde Dingsheng Test Machine Equipment Co., Ltd., situated in Chengde, Hebei, China).

### 2.11. Thermal Analysis

A comprehensive thermal analysis of HDL-4 EPS was carried out using an advanced thermal analyzer (Netzsch, Karlsruhe, Germany). This involved testing precisely 4 mg of EPS powder in an Al_2_O_3_ crucible. The examination included a temperature ramp from 25 °C to 800 °C at a constant rate of 10 °C/min, while maintaining an Ar gas flow rate of 50 mL/min. The resulting data were carefully documented to interpret the thermal behavior of EPS as the temperature increased.

### 2.12. Heavy-Metal-Chelating Activity

According to the method of Abid et al. [20], the atomic absorption spectrometer (iCE 3500 from Thermo Scientific, Boston, MA, USA) was utilized to evaluate the chelation capacity of HDL-4 EPS with Cu^2^⁺, Zn^2^⁺, Fe^2^⁺, Pb^2^⁺, and Cd^2^⁺ ions. A solution of 1 g/L HDL-4 EPS and a 0.8 g/L solution of metal ions were prepared and then combined in equal volume proportions (1:1, *v*/*v*), resulting in a concentration of 0.4 g/L for the metal ions and 0.5 mg/mL for the EPS. The mixture was allowed to react under ambient conditions for thirty minutes. Subsequently, the suspension was centrifuged at 6000 rpm for twenty minutes. A standard curve of metal ion concentration was produced, and the residual concentration of metal ions in the supernatant was determined.

### 2.13. Rheological Properties

The rheological properties of HDL-4 EPS were measured using a rheometer (HAAKE MARS60, Karlsruhe, Germany). The viscosities of HDL-4 EPS at different concentrations (20 mg/mL, 40 mg/mL, and 60 mg/mL) and pH levels (4, 6, and 8) were measured. The rotational speeds used in the test were 6 rpm, 60 rpm, and 100 rpm.

### 2.14. Emulsifying Activity (EA) Assay

According to the method of Yang et al. [17], equal volumes of 1.0 mg/mL HDL-4 EPS solution were mixed with hexane, benzene, xylene, petroleum ether, ether, gasoline, diesel oil, and soybean oil, respectively. After thorough mixing, the solutions were introduced into glass test tubes and allowed to stand at room temperature for 72 h. The height of the mixed liquid and emulsion layer was measured, and the emulsion activity (EA) was calculated using EA (%) = (M_1_/M_0_) × 100, where M_0_ is the height of the mixed liquid and M_1_ is the height of the emulsion layer.

### 2.15. Scavenging Assay of DPPH and ABTS Radical

According to the method described by Zhao et al. [18], 2 mL of HDL-4 EPS solution with concentrations ranging from 0 to 2 mg/mL was mixed with 0.2 mM DPPH–alcohol mixture, and the absorbance at 517 nm was measured after a 30 min incubation in darkness. In a separate experiment, 4 mL 7 mM ABTS working solution was combined with 0.2 mL of HDL-4 EPS solution at the same concentrations, and then incubated at 37 °C for 6 min. The absorbance at 734 nm was recorded, with vitamin C (Vc) used as the control. The scavenging activity percentage was calculated using the formula [(1 − (A_1_ − A_2_)/A_0_) × 100], where A_0_ represents the absorbance of the free radical in the absence of the sample, while A_1_ and A_2_ represent the absorbance readings when the sample interacts with the free radical and deionized water, respectively.

### 2.16. Probiotic Proliferation Test

Referring to the method of Yu et al. [15], *Lactobacillus plantarum* and *Streptococcus thermophilus* were inoculated into the culture medium and cultured at a constant temperature of 30 °C for 48 h. The activated two strains were inoculated into MRS culture medium with HDL-4 EPS, glucose, and inulin as single carbon sources, and samples were collected at 6, 12, 18, 24, and 36 h, the quantity of viable bacteria in sample was calculated based on the dilution multiple [18]. The sample was diluted using a gradient of sterile water. After dilution to a suitable concentration, 1 mL of diluted sample was applied to MRS solid medium at 30 °C for 48 h, and the quantity of colonies on the medium was recorded.

### 2.17. Water Solubility Index (WSI) and Water Holding Capacity (WHC) Analysis

Based on the approach of Liu et al. [21], the WSI of EPS proceeded as follows. A total of 45 mg of EPS was dissolved in 0.5 mL distilled water and subjected to vortex mixing for a duration of 2 h to ensure a consistent suspension. Insoluble EPSs were then separated via centrifugation at 10,000 rpm for 30 min at 4 °C. The resulting precipitate was freeze-dried and its mass recorded. The WHC evaluation followed a standard protocol. Specifically, 30 mg of EPS was evenly dispersed in 0.5 mL of MilliQ water, and the mixture was allowed to equilibrate at 30 °C for half an hour. A subsequent centrifugation step was carried out at 10,000 rpm for 30 min at 4 °C. After that, the remaining insoluble portion was blotted dry using filter paper. The WHC was calculated using the formula WHC(%) = (M_1_/M_2_) × 100, where M_1_ represented the initial weight and M_2_ denoted the weight of the freeze-dried residue.

### 2.18. Statistical Analysis

All tests were independently conducted three times. The data were analyzed using JMP software (version 10.0.2) and presented as the mean ± standard deviation. Origin 2023 was utilized for generating graphs. Statistical analysis involved the use of analysis of variance (ANOVA) and Tukey tests, with a significance level of *p* < 0.05 indicating a meaningful difference.

## 3. Results and Discussion

### 3.1. Strain Identiffcation, Extraction, and Quantification of HDL-4 EPS

**Figure 1:**
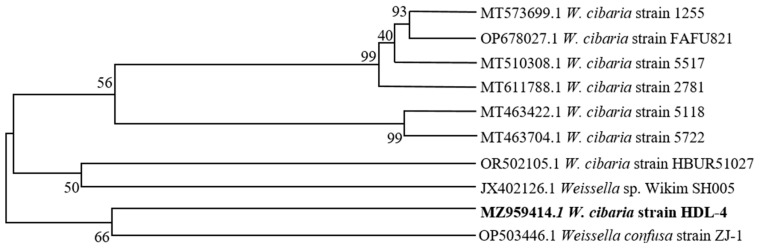
NJ tree representing the phylogenetic relationship based on 16 s rDNA gene sequences. (The bold indicated the identified strain.)

Based on morphological attribute analysis and 16S ribosomal DNA sequencing, the strain HDL-4 was identified as a member of the *W. cibaria* species. This species was characterized by positive Gram staining and a rod-like shape. The NCBI GenBank currently houses the sequenced data with the accession reference MZ959414. In order to conduct a comparative examination, a phylogenetic tree was constructed using the 16S rRNA sequences of *Weissella* and its associated taxa (Figure 1).

**Figure 2:**
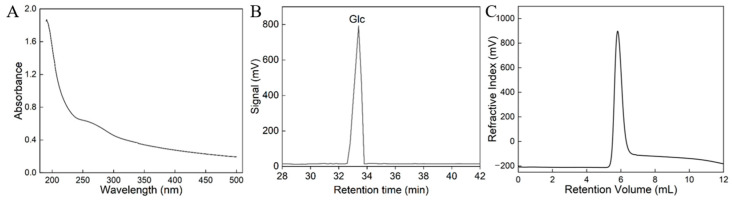
UV-Vis spectrum (**A**), GC chromatogram (**B**), and GPC chromatogram (**C**) of the HDL-4 EPS.

The production yield of crude exopolysaccharide (EPS) from *W. cibaria* HDL-4 was 28.38 ± 1.86 g/L. Following chromatographic separation via a Sephadex G-100 column, the most abundant polysaccharide fraction, HDL-4 EPS, was isolated, resulting in a prominent EPS peak. As shown in Figure 2A, the analysis of the UV spectra revealed a single peak in the range of 190 to 210 nm, which was characteristic of carbohydrates. The absence of signals at 260 and 280 nm indicated that the sample did not contain proteins or nucleic acids [22]. Protein content determination and chemical composition analysis showed that HDL-4 EPS only contains carbohydrates, with no detected content of proteins. This result was in accordance with the finding of Liu et al. [21] regarding the purification of *Saccharomyces cerevisiae* Y3 EPS. The purity of HDL-4 EPS has been preliminarily confirmed and further verified through GPC.

### 3.2. Monosaccharide Composition and Mw Analysis

As shown in Figure 2B, the GC analysis of HDL-4 EPS revealed the presence of glucose exclusively, suggesting that HDL-4 EPS was a homogeneous polysaccharide composed entirely of glucose units. This correspondence with the predominant monosaccharide, glucose, was consistent with the previously reported monosaccharide profile of EPS from *Weissella* species [23,24]. Additionally, certain strains of *Weissella* were known to produce fructan, glucan, and galactan type EPSs [25]. The biological activities of LAB EPSs were intricately linked to their structural attributes, which included the composition of monosaccharides and glycosidic linkages. EPSs with a high glucose content have a strong immune-stimulating effect [26].

The Mw of EPS was determined using GPC analysis. As shown in Figure 2C, the chromatogram exhibited a single peak, providing evidence of the purity of the HDL-4 EPS. The Mw of HDL-4 EPS was calculated to be 1.9 × 10⁶ Da. In contrast, previous research has reported that the EPS derived from *Weissella confusa* MD1 had a significantly lower Mw of 2.909 kDa [27]. Additionally, research by Ozpinar et al. [28] revealed that under MRS medium with 30 g/L sucrose, the Mw of the EPS synthesized by *W. confusa* S6 was 8 × 10⁶ Da. The Mw of these EPSs varied depending on the bacterial strain, cultivation conditions, and culture medium constituents. Understanding the Mw is crucial for elucidating the structure–function correlation of EPS. Observations suggest that biological activity is influenced by EPSs’ Mw, with a higher Mw resulting in increased viscosity in aqueous solutions and exhibiting potent anticancer properties, while lower Mw EPSs are recognized for their enhanced antioxidant activities [29,30].

### 3.3. FT-IR Analysis

**Figure 3:**
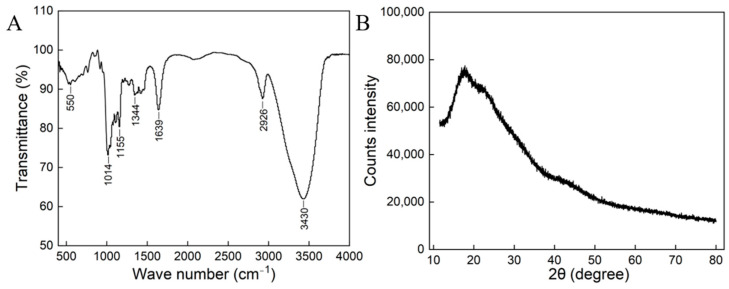
FT−IR spectrum (**A**) and XRD spectra (**B**) of the HDL-4 EPS.

According to Figure 3A, the FT-IR transmission absorption spectrum of HDL-4 EPS is in the range of 4000-400 cm^−1^, with a strong peak at 3430 cm^−1^ that indicates the stretching vibration of O-H in EPS [31]. It was determined that the peak at 2926 cm^−1^ resulted from the stretching vibration of C-H bonds and CH_2_ bonds [32], while the peak at 1639 cm^−1^ resulted from the stretching vibration of the sugar ring C=O [33]. The absorption peaks within the range of 1000-1200 cm⁻^1^ were ascribed to the stretching vibrations of C-O-C and C-OH groups, which were associated with the pyranose ring in polysaccharides. The extension of C-O-C and C-O atoms were detected in the range of 950–1100 cm^−1^, affirming the polysaccharide composition of the analyzed polymers [34]. The peak near 1014 cm^−1^ signified α-configuration [35]. Specifically, the characteristic peak at 1014 cm^−1^ corresponded to the (1*→*6)-α-D-glucose unit in the polysaccharide structure [36,37]. 

### 3.4. XRD Analysis 

The XRD spectrum revealed a broad peak near 19° (2θ) (Figure 3B), indicating that the EPS was amorphous. Similar studies have shown that EPSs produced by *W. confusa* C19, *Bacillus cereus* KMS3-1, and *W. confusa* XG-3 also exhibited amorphous characteristics [20,38,39].

### 3.5. NMR Analysis

**Figure 4:**
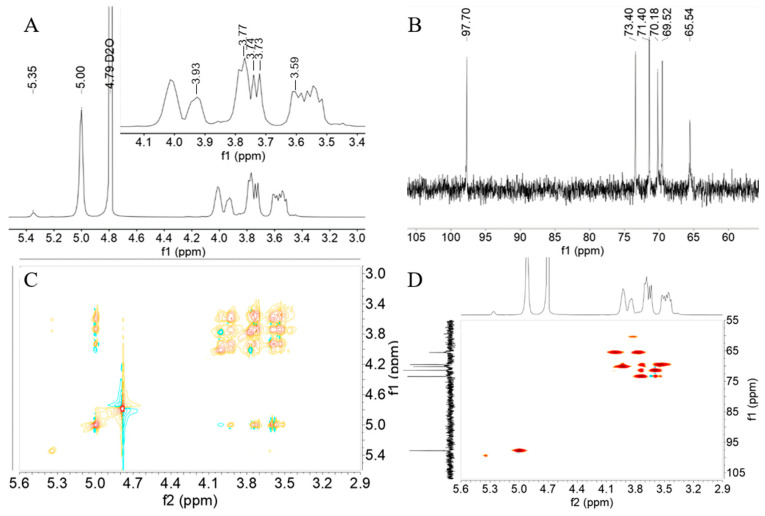
**^1^**H (**A**), ^13^C (**B**), COSY (**C**), and HSQC (**D**) NMR spectrum of HDL-4 EPS.

**Figure 5:**
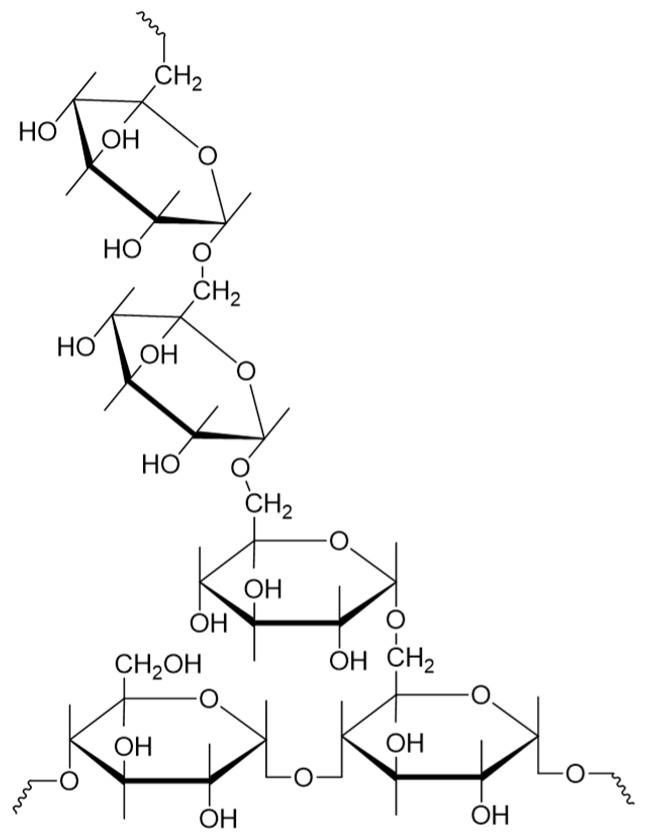
The predicted structural formula of HDL-4 EPS.

The glycosidic bond characteristics in the EPS structure were analyzed using ^1^H and ^13^C NMR. As shown in Figure 4A, spectral resonance between 3.1 and 5.4 ppm was observed in the ^1^H spectrum of EPS [40]. The strong peak at 4.79 ppm originates from HDO, while other peaks, recorded as overlapping signals between 3.4 ppm and 4.2 ppm, correspond to other protons in the EPS structure [23]. This finding indicates that EPS was a glucan, primarily composed of (1*→*6)-linked α-d-glucose units, with 3.13% (1*→*4)-linked α-d-glucose units. In the ^13^C NMR spectrum (Figure 3B), six signals at 65.54 ppm, 70.18 ppm, 69.52 ppm, 73.40 ppm, 71.40 ppm, and 97.70 ppm were observed, corresponding to the positions of C-6, C-5, C-4, C-3, C-2, and C-1 of EPS, respectively [41]. For 2D NMR analysis, HSQC and COSY data were recorded, and the intricate and overlapping information confirmed the relationship between protons and their corresponding carbons at 5.00/97.70 (H1/C1), 3.59/71.40 (H2/C2), 3.74/73.40 (H3/C3), 3.73/69.52 (H4/C4), 3.93/70.18 (H5/C5), and 3.77/65.54 (H6/C6), confirming the structure of EPS as a glucan through the correlation between ^1^H and their corresponding ^13^C spectra. As shown in Figure 5, ^1^H, ^13^C NMR, and 2D NMR data confirmed that EPS contained continuous (1*→*6)-linked α-D-glucose units and (1*→*4)-linked α-D-glucose units. The production of dextran EPS was a typical feature of some LABs, though the synthesis of dextran with low levels of (1*→*4)-linked α-D-glucose units can vary among different lab strains. For example, *Leuconostoc mesenteroides* B-742 produced dextran containing 13% (1*→*4)-linked α-D-glucose units. Compared to (1*→*3)-linked α-D-glucose units, these branches increased dextran’s resistance to endoglucanase hydrolysis [42]. Many *Lactobacillus reuteri* strains produced reuteran-type dextran, primarily formed by (1*→*4)-linked α-D-glucose units [43,44]. The EPS is an α-D-glucan containing (1*→*6) and 3.13% (1*→*4)-linkages, which may play an important role in different fields. Studies have indicated that the presence of β-1,3 glycosidic bonds in EPS and β-1,6 glycosidic bonds in its branches were closely associated with its anti-tumor activity [45]. EPS produced by *Bacillus amyloliquefaciens* is a type of α-glucan, consisting of α-(1*→*3) and α-(1*→*6) glycosidic bonds. It possesses the capability to scavenge superoxide anions [46].

### 3.6. SEM Analysis

**Figure 6:**
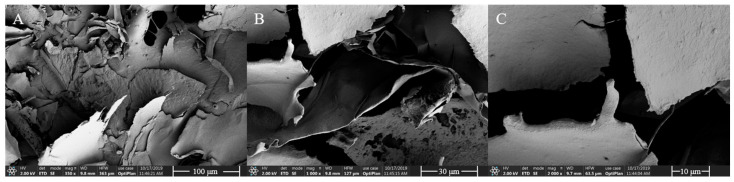
SEM images of HDL-4 EPS at 350× (**A**), 1000× (**B**), and 2000× (**C**) magnification.

The surface properties of HDL-4 EPS were studied using SEM, providing insights into its physical properties. At low magnification (350×), HDL-4 EPS presented a layered structure with pores and a smooth, bright surface (Figure 6A), resembling the polymer properties reported by Ye et al. [47]. These characteristics indicated that the EPS can form polymers with other substrates to enhance product performance, such as increasing water-holding capacity and viscosity, making it suitable as a thickener and gel in the food and cosmetics industries [48]. At higher magnification (1000× and 2000×) (Figure 6B,C), the surface appeared smooth and compact, similar to materials used for plasticized films [49]. However, this differs from the layered network structure with pores seen in *W. confusa* R003 EPS [50]. These differences among polysaccharide polymers may be attributed to variations in the strain source, monosaccharide composition, and extraction methods [51].

### 3.7. AFM Analysis

**Figure 7:**
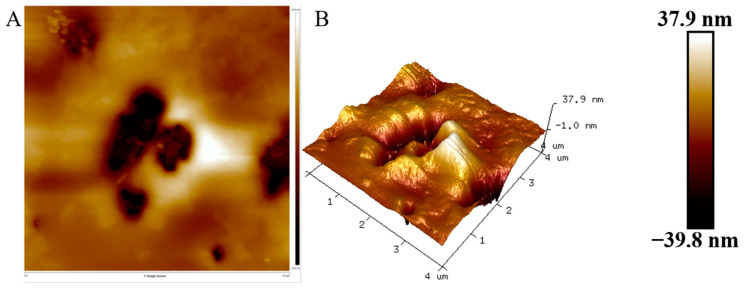
AFM images of HDL-4 EPS: planar view (**A**) and cubic view (**B**).

The polymers’ microstructure was analyzed using AFM. The AFM image of the HDL-4 EPS solution revealed an uneven and rough surface with irregular protrusions, with a maximum height of 37.9 nm (Figure 7). The length of hundreds of nanometers indicated aggregation of the glucomannan chains. HDL-4 EPS demonstrated an exceptional ability to attract water due to its tightly packed molecular arrangement. Its molecular structure resembled the EPS synthesized by *Lactobacillus kefiranofaciens*, as previously observed by Ahmed et al. [52]. The morphological findings from AFM images highlighted the superior hydrophilic nature of EPS, which was a crucial characteristic for its large-scale production and technological applications.

### 3.8. Water Contact Angle Analysis

**Figure 8:**
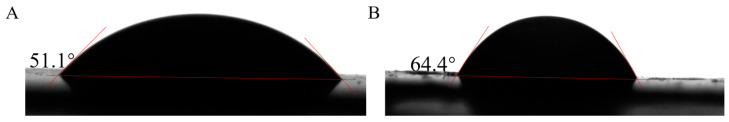
Water contact angle analysis of HDL-4 EPS in MRS at 15 s (**A**) and MRS +5% sucrose at 15 s (**B**).

The assessment of bacterial EPS hydrophobicity often involves measuring the water contact angle. As shown in Figure 8, HDL-4 EPS exhibits a significant increase in this angle, rising from 51.1° in standard MRS medium to 64.4° when grown with 5 g/L sucrose. This increase suggested an enhanced hydrophobic nature of HDL-4 EPS’s surface. Similar results were observed with EPS from *W. confusa* XG-3 [20] and *Lactobacillus sakei* L3 [53].

### 3.9. Thermal Properties Analysis

In the TGA curve, as the temperature increased, HDL-4 EPS mainly underwent gelation and swelling, followed by dehydration and cracking at higher temperatures [54]. The TGA curve described the weight reduction in HDL-4 EPS at 40–800 °C, which can be divided into three stages. Between 40 and 90 °C, the weight of HDL-4 EPS decreased by about 9%, likely due to water loss. Similarly, the EPS of *W. confusa* S6 [28] had a small weight loss at 100 °C. Between 90 °C and 288 °C, the weight of HDL-4 EPS remained relatively stable, indicating stability below 288 °C. From 288 °C to 407 °C, the weight of HDL-4 EPS decreased significantly (61%), likely due to depolymerization. This was consistent with the results for *L. lactis* L2 EPS, which also showed a sharp weight drop between 256 and 392 °C [19]. The DTG curve indicated two steps in HDL-4 EPS degradation: an initial peak at 48 °C related to bound water loss, and another peak at 308 °C where the weight loss rate was highest, higher than that of EPS from *Leuconostoc mesenteroides* DRP105, which degrades at 279 °C [55]. The weight of HDL-4 EPS did not change significantly beyond this temperature, likely due to its complex molecular structure, including monosaccharide composition and chemical groups. The DSC curve showed the heat absorption and dissipation of EPS samples during the temperature increase. An endothermic melting peak at 308 °C can be attributed to the depolymerization of EPS, with the melting of long fatty side chains forming crystals, consistent with the TGA and DTG curves. These results demonstrate that HDL-4 EPS has a good thermal stability, with a degradation temperature much higher than those used in food processing. Therefore, HDL-4 EPS can be widely used in the food industry.

### 3.10. Heavy-Metal-Chelating Activity of HDL-4 EPS

**Figure 9:**
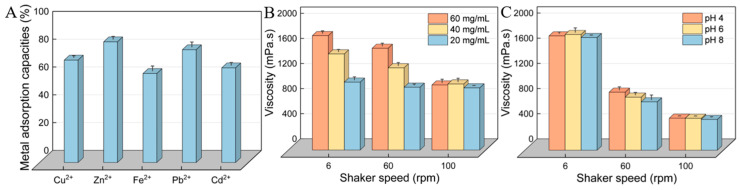
(**A**) Metal adsorption activity. (**B**,**C**) Rheological properties: (**B**) different concentrations of EPS and (**C**) different pH values of HDL-4 EPS.

It has been reported that compounds containing functional groups such as -OH, -O-, -COOH, -PO_3_H_2_, -SH, and -S- exhibit metal chelating abilities [56]. Consequently, EPS shows potential for removing heavy metal pollution. As demonstrated in Figure 9A, HDL-4 EPS exhibited high chelation capacities for Cu^2+^, Zn^2+^, Fe^2+^, Pb^2+^, and Cd^2+^, with scavenging capacities of 73.7%, 86.9%, 64.1%, 81.2%, and 68.1%, respectively. Compared to the previously reported *Leuconostoc mesenteroides* HDE-8 EPS, HDL-4 EPS demonstrated superior Pb^2+^ scavenging ability. However, its chelation efficacy for Cu^2+^ and Fe^2+^ was lower than that of HDE-8 EPS, which had adsorption rates for Pb^2+^, Cu^2+^, and Fe^2+^ of 77.62%, 82.84%, and 87.56% [17], respectively. The adsorption rate of Fe^2+^ by HDL-4 EPS surpassed that of *Bacillus amyloliquefaciens* GSBa-1 EPS, which was 30.5% [46]. The metal adsorption activity of HDL-4 EPS might be attributed to its abundant -OH and -COOH [57]. Similarly, a study has demonstrated that (1→3)-α-glucan derived from *Lentinus edodes* exhibits a high metal adsorption activity [58]. HDL-4 EPS is a potent metal adsorbent and is emerging as an effective and readily available biological adsorbent. It has the potential to mitigate heavy metal pollution in the environment and food, and it is expected to be widely utilized in environmental and food industries.

### 3.11. Rheological Property of HDL-4 EPS

The study of rheological properties is crucial for analyzing the physical properties of EPS and provides a theoretical basis for its applications in food and medicine. Figure 9B,C showed that the viscosity of HDL-4 EPS gradually decreases with increasing rotational speed, likely due to the reduction in intermolecular forces under higher shear forces. As a result, the intermolecular distance increased, leading to decreased friction and adsorption forces between EPS molecules, ultimately reducing viscosity. Additionally, the viscosity of EPS decreased in a dose-dependent manner, similar to the trend observed for EPS produced by *Lactiplantibacillus plantarum* HDC-01 [15]. Figure 9C also indicated that the viscosity of HDL-4 EPS was relatively high in neutral solutions, which was consistent with previous reports. This may be due to the combination of hydroxide and hydrogen ions with hydroxyl and carboxyl groups under acidic and alkaline conditions, resulting in decreased intermolecular forces and reduced viscosity [17].

### 3.12. EA Activity of HDL-4 EPS

Emulsifiers are crucial substances that promote the formation of stable emulsions between different components. Currently, biological emulsifiers, which are non-toxic, natural, and biodegradable, have garnered significant attention from researchers. The emulsification properties of EPS are attributed to the presence of hydrophilic and hydrophobic functional groups in the molecule [59]. As shown in Table 1, HDL-4 EPS exhibited high emulsifying activity for petroleum ether and soybean oil. After 48 h, the emulsification rates reached 23.73 ± 1.18% and 36.84 ± 0.98%, respectively, which were significantly higher than those of *Leuconostoc citreum* B-2 EPS (9.01 ± 2.44%) [60]. However, the emulsifying activity of HDL-4 EPS for n-hexane was low, with an emulsification rate of 6.18 ± 0.20% at 48 h. Meanwhile, the emulsifying activity of HDL-4 EPS for various organic solvents increases. HDL-4 EPS has strong emulsifying properties and can be used as an additive to promote the water-oil balance in cosmetic emulsions. Thus, it can be utilized in the food industry to improve the texture of cream and ice cream.

### 3.13. DPPH Radical and ABTS Radical Scavenging Activity of HDL-4 EPS

**Figure 10:**
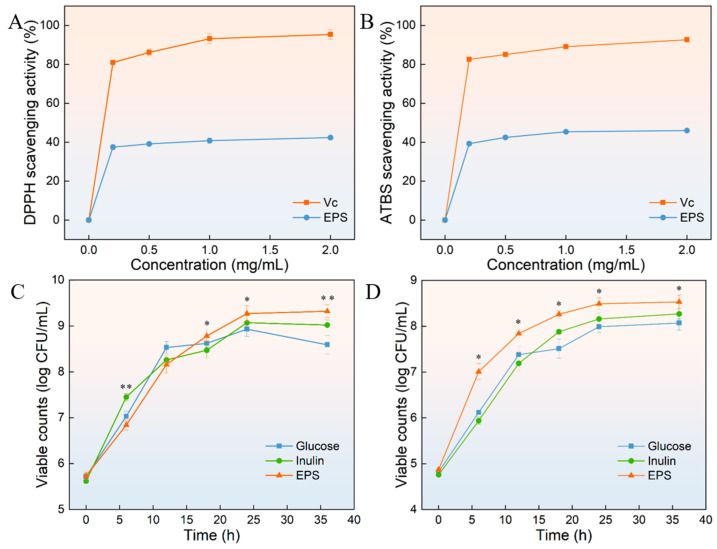
The scavenging activity of (**A**) DPPH radicals and (**B**) ABTS radicals of HDL-4 EPS with Vc as the positive control and the proliferation effect of HDL-4 EPS and commercial prebiotics (inulin and glucose) on probiotics (*L. plantarum* (**C**) and *S. thermophilus* (**D**)). The asterisks denote a significant difference in the probiotic proliferation of commercial prebiotics and HDL-4 EPS (*p* < 0.05).

DPPH is an effective reagent to evaluate the activity of antioxidants in scavenging free radicals. Antioxidants can release hydrogen ions, thereby reducing the purple DPPH radical. The final solution was yellow and showed a strong absorption peak at 517 nm [61]. Figure 10A illustrated the scavenging activity of Vc and HDL-4 EPS against DPPH radicals. Notably, HDL-4 EPS demonstrated a significantly lower scavenging activity compared to the Vc group (*p* < 0.05). The DPPH radical scavenging activity of HDL-4 EPS was 37.5 ± 1.3%, 39.1 ± 0.8%, 40.8 ± 1.4%, and 42.3 ± 0.4%, when the HDL-4 EPS concentration was 0.2 mg/mL, 0.5 mg/mL, 1 mg/mL, and 2 mg/mL, respectively. The result showed that the DPPH radical scavenging activity was concentration-dependent between 0 mg/mL and 0.2 mg/mL. The DPPH radical scavenging activity of HDL-4 EPS did not increase significantly (*p* < 0.05) when the EPS concentration was higher than 0.2 mg/mL. The DPPH radical scavenging activity of HDL-4 EPS was higher than *Streptomyces* sp. MOE6 EPS (37.8 ± 0.1%) [62] but lower than that of *Bacillus megaterium* PFY-147 EPS with a scavenging activity of 70% [63]. EPS containing functional groups such as hydroxyl and carboxyl could enhance its DPPH radical scavenging activity. Differences in Mw and monosaccharide composition also contributed to the DPPH and ABTS radical scavenging activity of different EPSs [64]. Similarly, the ATBS radical was used to evaluate antioxidant capacity due to its relative stability. As shown in Figure 10B, the ATBS radical scavenging activity of HDL-4 EPS against ATBS radicals was lower than that of the Vc group. The ATBS radical scavenging activity of HDL-4 EPS was 39.3 ± 1.0%, 42.5 ± 1.1%, 45.3 ± 0.9%, and 46.0 ± 0.2%, when the HDL-4 EPS concentration was 0.2 mg/mL, 0.5 mg/mL, 1 mg/mL, and 2 mg/mL, respectively. Similarly, when the concentration of HDL-4 EPS was between 0 mg/mL and 0.2 mg/mL, the ATBS radical scavenging activity was concentration-dependent. Compared with the HDL-4 EPS concentration of 0.2 mg/mL, when the EPS concentration was 0.5 mg/mL, there was no significant difference in the scavenging activity of ATBS (*p* < 0.05). HDL-4 EPS was consistent with the ATBS radical scavenging activity of *L. plantarum* KX041 EPS [65]. It can be seen that when the concentration of HDL-4 EPS was higher than 0.2 mg/mL, the antioxidant activity of EPS did not increase significantly. Therefore, EPS at this concentration can be used as an antioxidant in different fields.

### 3.14. Probiotic Proliferation

The probiotic effects of different prebiotics on two probiotics are shown in Figure 10C,D. It was found that various prebiotics can promote the growth of probiotics and shorten the logarithmic growth period of bacteria. Before 15 h, the probiotic effect of HDL-4 EPS on *L. plantarum* was lower than that of glucose and inulin. However, after 12 h, the probiotic effect of HDL-4 EPS was significantly stronger than that of other prebiotics (*p* < 0.05), stabilizing at 24 h. The probiotic effect of HDL-4 EPS on *L. plantarum* was stronger than that of EPS produced by *L. plantarum* HDC-01 [15]. Figure 10D demonstrated that the probiotic effect of HDL-4 EPS on *Streptococcus thermophilus* was significantly stronger than that of glucose and inulin at different time points (*p* < 0.05). This finding suggests that HDL-4 EPS had a greater ability to promote the growth of *S. thermophilus* compared to glucose and inulin. These results indicated that HDL-4 EPS had obvious beneficial effects on different probiotics. The proliferation effect of HDL-4 EPS on probiotics was similar to that of EPS produced by *W. confusa* XG-3 [18]. Compared with other prebiotics, HDL-4 EPS was better utilized by the strain and promoted growth more effectively. Further studies are needed to explore the beneficial effects of different concentrations of HDL-4 EPS on probiotics.

### 3.15. WSI and WHC Analysis of HDL-4 EPS

The WSI measurement of HDL-4 EPS was recorded at 96.57 ± 1.03%, with a WHC of 432.92 ± 4.38%, exceeding both the EPSs derived from *Leuconostoc pseudomesenteroides* and *L. lactis* [66,67]. The high WHC of the water-soluble EPS was attributed to its absorbent architecture, which can retain substantial water through hydrogen bonding. Previous research has shown that the WHC of yogurt significantly increased when prepared using a co-culture of EPS-producing bacteria as a starter. These findings suggest that EPS possesses viscoelastic properties, such as water solubility and various forms of solubility, which contribute to enhancing the texture and flow behavior of food products.

## 4. Conclusions

In this study, EPS produced by *W. cibaria* HDL-4, isolated from litchi, was purified, and its structure and properties were analyzed. HDL-4 EPS, with a molecular weight of 1.9 × 10^6^ Da, was a D-type glucan composed of glucose with α-(1→6) and α-(1→4) glycosidic bonds. The morphology of HDL-4 EPS presents a layered structure with pores, slight protrusions, and a smooth surface. The physical and chemical properties of HDL-4 EPS were evaluated, showing good thermal stability, emulsification activity, and rheological properties, while also having a good ability to stimulate the growth of probiotic bacteria. These attributes make it a natural, safe, and ideal additive in the dairy industry. Additionally, HDL-4 EPS demonstrates high metal adsorption activity, strong water-retaining property, and high antioxidant activity, which supports its application in medicine, the chemical industry, and other fields. In summary, the results reveal the great potential of HDL-4 EPS in the food, medicine, and chemical industries, providing a theoretical basis for the application of EPS in various fields.

## Figures and Tables

**Table 1:** EA% of HDL-4 EPS with organic agents.

Organic Agents	E_24_ (%)	E_48_ (%)
Hexane	5.57 ± 0.28	6.18 ± 0.20
Benzene	12.30 ± 0.91	17.3 ± 0.57
Petroleum ether	15.65 ± 0.81	23.73 ± 1.18
Gasoline	15.97 ± 0.65	20.70 ± 0.86
Soybean oil	26.80 ± 0.78	36.84 ± 0.98

## Data Availability

Data are contained within the article.
